# Biomechanical modeling as a practical tool for predicting injury risk related to repetitive muscle lengthening during learning and training of human complex motor skills

**DOI:** 10.1186/s40064-016-2067-y

**Published:** 2016-04-12

**Authors:** Bingjun Wan, Gongbing Shan

**Affiliations:** School of Physical Education, Shaanxi Normal University, Xi’an, China; Department of Kinesiology, University of Lethbridge, 4401 University Drive, Lethbridge, AB T1K 3M4 Canada; Department of Physical Education, Xinzhou Teachers’ University, Xinzhou, Shanxi China

**Keywords:** 3D motion capture, ROM, Over-lengthening, Impact-like eccentric muscle tension

## Abstract

Previous studies have shown that muscle repetitive stress injuries (RSIs) are often related to sport trainings among young participants. As such, understanding the mechanism of RSIs is essential for injury prevention. One potential means would be to identify muscles in risk by applying biomechanical modeling. By capturing 3D movements of four typical youth sports and building the biomechanical models, the current study has identified several risk factors related to the development of RSIs. The causal factors for RSIs are the muscle over-lengthening, the impact-like (speedy increase) eccentric tension in muscles, imbalance between agonists and antagonists, muscle loading frequency and muscle strength. In general, a large range of motion of joints would lead to over-lengthening of certain small muscles; Limb’s acceleration during power generation could cause imbalance between agonists and antagonists; a quick deceleration of limbs during follow-throughs would induce an impact-like eccentric tension to muscles; and even at low speed, frequent muscle over-lengthening would cause a micro-trauma accumulation which could result in RSIs in long term. Based on the results, the following measures can be applied to reduce the risk of RSIs during learning/training in youth participants: (1) stretching training of muscles at risk in order to increase lengthening ability; (2) dynamic warming-up for minimizing possible imbalance between agonists and antagonists; (3) limiting practice times of the frequency and duration of movements requiring strength and/or large range of motion to reducing micro-trauma accumulation; and (4) allowing enough repair time for recovery from micro-traumas induced by training (individual training time). Collectively, the results show that biomechanical modeling is a practical tool for predicting injury risk and provides an effective way to establish an optimization strategy to counteract the factors leading to muscle repetitive stress injuries during motor skill learning and training.

## Background

### Risk of muscle injury linked to human complex motor skills’ acquisition

The main focuses of human movement and performance research have been to improve and accelerate skill acquisition (education and training), maintain motor function (daily living and activities), and at the same time reduce occurrence of injury among those participating in the activities (health and wellness) (Ballreich and Baumann 1996; Shan et al. 2004a; Visentin et al. 2008; Winter 1990). The desirability of acquiring motor skills efficiently and effectively while simultaneously avoiding injury would seem self-evident. Consequently, researchers, professional educators, medical practitioners and participants are becoming more occupied with such matters, especially in the motor learning and training of complex skills among young participants (American Academy of Pediatrics Committee on Sports Medicine and Fitness 2000; Axe et al. 2001; Zaremski and Krabak 2012).

Complex human motor skills, such as throwing techniques, various kicks in dancing and sports, are of learned human behaviors. The motor control of these skills is so complicated that it requires a harmonized effort of the whole-body, multi-joint coordination—an entire kinetic chain control (Shan et al. 2015; Shan and Westerhoff 2005; Visentin et al. 2015). Such behaviors require intricate motor control, perception and adaptation in a temporal endeavor (Shan et al. 2012); and the motor learning needs years’ repetitive training or practice, starting at a young age. One possible negative outcome of such long-time repetitive training/practice is overuse syndrome (OS) (Burkhart et al. 2003; Reid et al. 1987; Sohl and Bowling 1990). Muscle injury caused by repetitive muscle lengthening during learning and training is one type of OS.

Muscle injuries from physical activity fall into two main categories—impact injuries where the physiological limits of the individual are surpassed (Peterson and Renström 1987) and repetitive stress injuries (RSIs) where the accumulation of micro-injury from physical overuse surpass the individual’s physiological tolerance (Dawson et al. 1990; Visentin and Shan 2004). While the mechanism of impact injuries is well studied, RSIs represent a different line of inquiry (Shan et al. 2004b). RSIs are typical of many physical activities, such as sports, dancing and playing musical instruments (Visentin and Shan 2004). Since RSIs can be linked to learned motor behaviors, their etiology must be examined with a view to quantifying factors associated with the development of injury in order to have the potential to influence these behaviors.

### Repetitive stress injuries and school sports

A closer look at current North American school sport reveals an alarming statistic, over 70 % of participants drop out of youth sport programs along the way to high school and the 4th cause of youth dropouts in sport is an increased injury incidence due to inordinate demands on young bodies (Woods 2011). Muscle strains (pulled muscles)—one type of RSIs—are among the most common injuries in sports—as many as 30 % of the injuries seen in sports medicine are muscle strain injuries (Garrrett 2002). These strains usually occur during sprinting, kicking, stretching, overhead throwing and rapid stop/deceleration (Garrrett 2002; Oyama 2012; Shan 2005; Zaremski and Krabak 2012). The situation in school physical education could be even worse in Asia/China, yet there is no statistical evidence.

Effective human motor skill learning/training benefits nearly every one of us, as it can help develop interests in more physical activities and lead to more active and healthy lifestyles (Chen and Ennis 2004; Lee et al. 1995; Paffenbarger Jr et al. 1993). The high drop rate in school sports would become one of the health and wellness concerns for future generations. In order to promote physical activities among young adults, measures for minimizing injury risks related to RSIs during sports participation should be taken. One potential method for the prevention, however, would be an identification the risk induced by various physical conditions/postures. Such identifications would help coaches and practitioners develop proper training programs to reduce the risk of RSIs during training/practice, while keeping participants enthusiastic towards sports.

### Principles of risk identification

Sports motor skills are diverse. For achieving delimiting results, two aspects should be considered: typical skills in most popular sports and the existing literature related to muscular injury in the sports.

#### Typical skills in popular school sports

In North American, the top ten sport participation among school boys and girls (age 6–18 years) are listed in Table 1 (Woods 2011). The sports listed cover both simple and complex motor skills. Usually, a complex one involves elements of multi-joints coordination and rapid acceleration and/or deceleration of body segments, such as powerful kicking, rapid over-head throwing and stretching (Fleisig et al. 1999; Shan and Westerhoff 2005). For improving the effectiveness of those skills, one needs long-time repetitive training and, therefore, is highly related to RSIs (Garrrett 2002; Oyama 2012; Shan 2005; Zaremski and Krabak 2012).

**Table 1:** Most frequent physical activities in North American schools (data from Woods 2011)

Rank	Boys		Girls	
	Physical activities	% Participation	Physical activities	% Participation
1	Basketball	71	Dancing	61
2	Football	65	Swimming/diving	56
3	Soccer	51	Basketball	55
4	Jogging/running	49	Jogging/running	53
5	Swimming/diving	48	Volleyball	47
6	Baseball/softball	48	Bowling	47
7	Bowling	48	Soccer	40
8	Weight training	42	Baseball/softball	38
9	Bicycling	33	In-line skating	33
10	Skateboarding	29	Camping/hiking	29

**Fig. 1:**
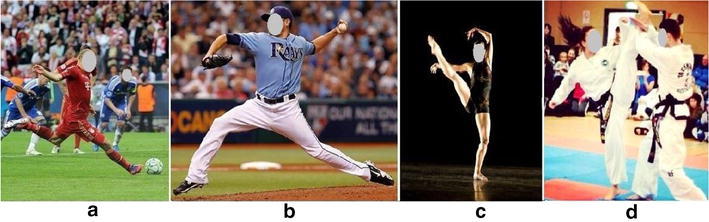
Complex motor skills selected from popular school sports for the study: **a** maximal instep kick, **b** baseball pitching, **c** fan kick, and **d** axe kick

Among boys’ top ten sports, maximal instep kick would be a typical skill (Fig. 1a), because this skill is often applied in football and soccer (the 2nd and the 3rd of boys’ sports). Additionally, girls’ participation rate of soccer is high too (40 %, the 7th). The complexity of the skill can be abstracted as the formation of a tension arc, and its fast release using a whip-like movement of the kick-leg (Shan et al. 2005; Shan and Westerhoff 2005). During the formation of a tension arc, certain muscles should be lengthened for generating an explosively powerful kick. The repetitive use of the skill during learning and training could induce leg muscles strain (Garrrett 2002)—a suitable case for studying risk of leg muscle injury.

A typical complex skill of upper-limbs would be baseball pitch (the 6th, also the 8th for girls) (Fleisig et al. 1999; Zaremski and Krabak 2012) (Fig. 1b). Although the most injured body area caused by baseball pitch is glenohumeral region (connective tissues surrounding the shoulder joint, a non-typical muscle injury) (Anderson and Alford 2010; Braun et al. 2009; Wilk et al. 2009), the Latissimus dorsi tears have been observed (Schickendantz et al. 2009). It is known that pectoralis major and latissimus dorsi are two main muscles involved in baseball pitching, quantification of muscle lengthening of these two muscles could reveal the injury mechanism related to the uniqueness of the skill.

The number one among girls’ top ten physical activities is dancing. Various stretching and kicking exercises outline dancing common practice. The fan kick, involving both stretching and kicking, is such a skill. The skill is wildly practiced and performed in various types of dance, such as ballet, modern and jazz dance. In a fan kick, the working leg makes a sweeping arc in front of the body and opens up to create a “fan” shape (Fig. 1c). Basically, the skill begins with a weight transfer to the supporting leg to help create a posture for initiating a circle movement of the working leg; the working leg is then to lift across the body to a peak height straight in front of the body and continues to swing out to the side of the body and downward until the weight is transferred onto the working leg. It is known that the rates of injuries in professional dancers are over 80 % (EMC 2003; Rovere et al. 1983), with 64–75 % of these injuries affecting the muscles and soft tissue (Arendt and Kerschbaumer 2003; EMC 2003). A quantitative characterization of muscle lengthening of the fan kick could help identify factors linked to the high injury rates.

#### The literature related to muscular injury and the aims of the current study

Previous studies have shown that the best time for preventing muscle strain injuries is at the beginning of learning and training, when good skill mechanics and good training habits can be developed (Andrews and Fleisig 1998; Arendt and Kerschbaumer 2003). A development of effective prevention program requires an understanding of the nature and cause of muscle strains. Usually, a muscle strain happens when a powerful muscle contraction is executed, combined with muscle over-lengthening (Garrrett 2002). Therefore, investigations focusing on body kinematics (range of motion (ROM), acceleration and/or deceleration) and their related muscles’ lengthening conditions in various complex motor learning and training situations would hold a great potential in developing injury prevention strategies in practice. Unfortunately, most previous studies are only related to injury-site examinations, statistical and/or epidemiological studies, and the observation of the behaviors associated with the injury—post injury study (Byhring and Bo 2002; McFarland and Wasik 1998; Quirk 1983; Register-Mihalik et al. 2011). They could not reveal the process of muscle injuries caused by overuse under real performance conditions and causal factors remain uncharted.

Further, empirical evidence has shown that movement variations and strength imbalance between agonists and antagonists play an important role in the etiology of these injuries (Bejjani 1987). Such evidence can also be characterized using ROM of joints and the change of muscles’ lengths (Visentin and Shan 2003). An examination of the relationship between these two type factors can be obtained by using 3D motion capture and biomechanical modeling. Therefore, the first aim of this study was to reveal the ROM characteristics and the dynamic muscles lengthening of the selected complex motor skills.

The causal factors revealed by animal experiments showed that a repetitive motion with 20 % more muscle lengthening than its rest length (i.e. 120 % of its rest length) would most likely cause muscle injuries (Faulkner et al. 1993; Fritz and Stauber 1988; McCully and Faulkner 1985; Stauber et al. 1988). But there is little study to characterize the dynamic change of muscle lengths during the performance of complex motor skills. Since young participants are at their early stage of training (relatively under-trained), repetitive muscle over-lengthening during learning and training could be dangerous and counter-productive. Therefore the second aim of this study was to identify muscles at risk (lengthening >120 % of its resting length) for the selected complex motor skills. This would help to establish preventive measures, which could be applied in learning and training programs.

The previous dance literature indicate that 64–80 % of dance injuries are in the lower extremities (Arendt and Kerschbaumer 2003; Milan 1994) and muscle strains and tears represent most of the injuries (Allen et al. 2012; Bejjani 1987; Murphy et al. 2003). From kinematic point of view (stretching), the relative leg-positions of the fan kick in dancing (Fig. 1c) and the axe kick in martial arts (Fig. 1d) are extremely alike. However, the leg muscle injury of martial artists is less than 10 %, much lower than that of dancers (Birrer and Halbrook 1988; Pieter and Zemper 1997; Wilkerson 1997). An effective way for initiating injury prevention strategy is to compare similar human activities with a significant difference in injury rate. Such a comparison could supply hints for injury prevention. As such, the third aim of this study was to compare kinematics between fan kick and axe kick in order to provide insight into risk assessment and muscle injury prevention in youth motor skill learning and training.

## Results and discussions

### Maximal instep soccer kicking

One of the aims of the study was to reveal the ROM characteristics and the dynamic lengthening of leg muscles during soccer kicking via 3D motion capture and biomechanical modeling. The aim was to explore to what degree the biomechanical modeling could identify muscles at risk and if the identification could be used to develop prevention strategies for motor learning and training in youth sports.

**Fig. 2:**
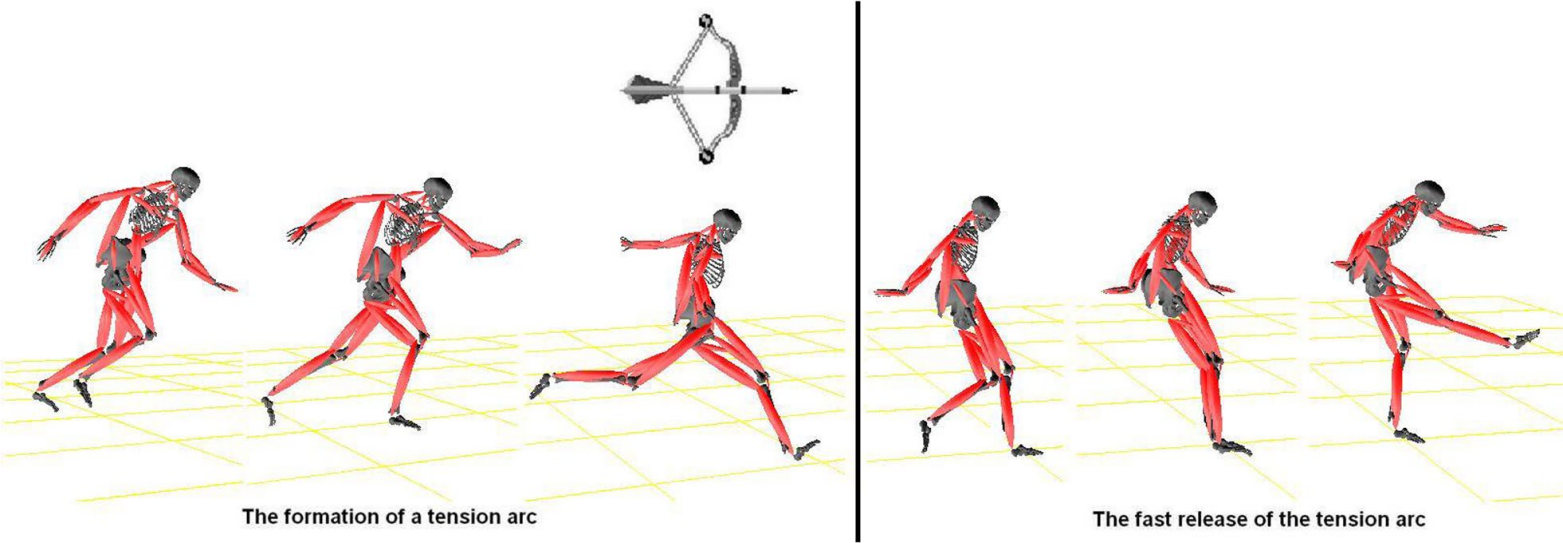
Biomechanical modeling of maximal instep kick in soccer

The results show that 3D motion capture and biomechanical modeling can be used to reveal distinguishable joints ROMs and the related muscles lengthening characteristics. The findings of the current study confirmed the existence of tension-arc phenomenon existed in skilled soccer players (Shan et al. 2005; Shan and Westerhoff 2005), i.e. the formation and the fast release of a tension arc during maximal instep kick (Fig. 2). Due to the formation of the tension arc, the kick-side hip experienced over-extension; as such, some leg muscles were over-lengthened. Since the kick-side hip over-extension and the whip-like movement of the kick leg mainly consisted of flexion/extension and adduction/abduction of hip and knee (Shan et al. 2005; Shan and Westerhoff 2005), the ROMs of the two joints and seven muscles responsible for the hip and knee movement were selected for the quantification. The selected muscles were hip flexor/knee extensor (rectus femoris and vastus laterialis of quadriceps), hip extensor (biceps femoris), hip adductors (gracilis and adductor magnus), hip abductor (gluteus medius), and a two-joint muscle contributing to hip extension and knee flexion (Semitendinosus). The results are shown in Table 2.

**Table 2:** Range of motion (ROM) of hip and knee as well as lengthening characteristics of selected muscles on the kick-side

ROM (°)		Muscle lengthening (% of its rest length)	
Hip			
Flexion/extension	129 ± 11	Rectus femoris	112.8 ± 8.4
Abduction/adduction	26 ± 5	Vastus laterialis	104.2 ± 6.1
Rotation	17 ± 4	Biceps femoris	119.3 ± 6.6
Knee			
Flexion/extension	113 ± 10	Adductor magnus	*174.6* ± *11.6*
Abduction/adduction	23 ± 6	Gracilis	*135.1* ± *8.6*
Rotation	19 ± 7	Gluteus medius	113.2 ± 6.9
		Semimembranosus	*127.7* ± *8.6*

Italics: muscles in risk

The 3D kinematic data reveals that the formation of the tension arc dramatically increases the ROM for hip and knee flexion/extension. The large ROMs cause some muscles’ lengthening over the critical value (i.e. 120 % of its rest length). The identified muscles in risk are adductor magnus, gracilis and semimenbranosus (Table 2). Repetitively practice of the formation of the tension arc would induce a risk of RSIs for young players. Among the three muscles, the Adductor Magnus is especially prone to injury, with its length value of 174.6 %. Since the formation of the tension arc is a key factor for increasing the kick power (Shan et al. 2005; Shan and Westerhoff 2005), its training is inevitable in practice. It has been hypothesized that, for beginners, flexibility training before the kick practice can help reduce the risk of RSIs.

Medical documentation has shown that one of the most common leg RSIs in soccer is muscle or tendon strain, which occur over time due to stress on the muscles (including soft tissues) without sufficient time for self-healing and/or an imbalance between agonists and antagonists (Garrrett 2002). The results of this study (i.e. the large ROMs of both the hip and the knee) would suggest that the strain and the imbalance between agonists and antagonists could happen during repetitive over-lengthening of muscle fibers in an improper training program. Therefore, for injury prevention during soccer learning and training, coaches and trainers should pay special attention to the following aspects: (1) stretching training of muscles at risk, (2) dynamic muscle warm-up before practice, (3) limiting practice times of repetitive kicking training, and (4) individual training time. The first aspect would increase muscle lengthening ability. Well trained muscles could reach 180 % of its rest length without injury (Wilkerson 1997). The second one would ascertain that muscle tightness can be minimized; as such the coordination efficiency between agonists and antagonists will be improved. The third one would slow down the rate of micro-trauma accumulation—a key cause of RSIs (Visentin and Shan 2004). The last one will allow individual to have enough repair cycles during periods of relative inactivity in order to allow a self-healing of the micro-trauma accumulated (Visentin and Shan 2004). Unlike impact injuries where there is virtually no time between loading and injury, repair cycles play an import role in prevention of RSIs. Coaches and trainers should be aware that the development of RSIs depends on a net of two biological processes—microtrauma accumulation and repair cycles (Visentin and Shan 2004) and if the microtrauma accumulation is faster than the repair cycles, muscle soreness will occur, resulting additionally in imbalance between agonists and antagonists (Bejjani 1987; Schickendantz et al. 2009). The best prevention would be to keep a dynamic balancing between microtrauma accumulation and repair cycles. In doing so, individual training time is required in practice, as individual muscle soreness requires different recovery time.

### Baseball pitching

The aim of the baseball pitching study was to investigate the possible injury mechanism of latissimus dorsi via biomechanical modeling. Previous studies indicated that seven critical events divided pitching into six phases (Fleisig et al. 1999; Nissen et al. 2007). The seven events are initial position, balance point, foot contact, maximum shoulder external rotation, ball release, maximum shoulder internal rotation, and fielding position. The six phases include wind-up, stride, arm cocking, arm acceleration, arm deceleration, and follow-through. The whole motion requires proper coordination of muscle activities in the trunk. It is known that the main trunk muscle in the forward movement of the arm is the pectoralis major and, towards the end of pitching, the arm has to be slowed down and thus, the back muscles (e.g. latissimus dorsi) become important (Nissen et al. 2007; Park et al. 2002).

**Fig. 3:**
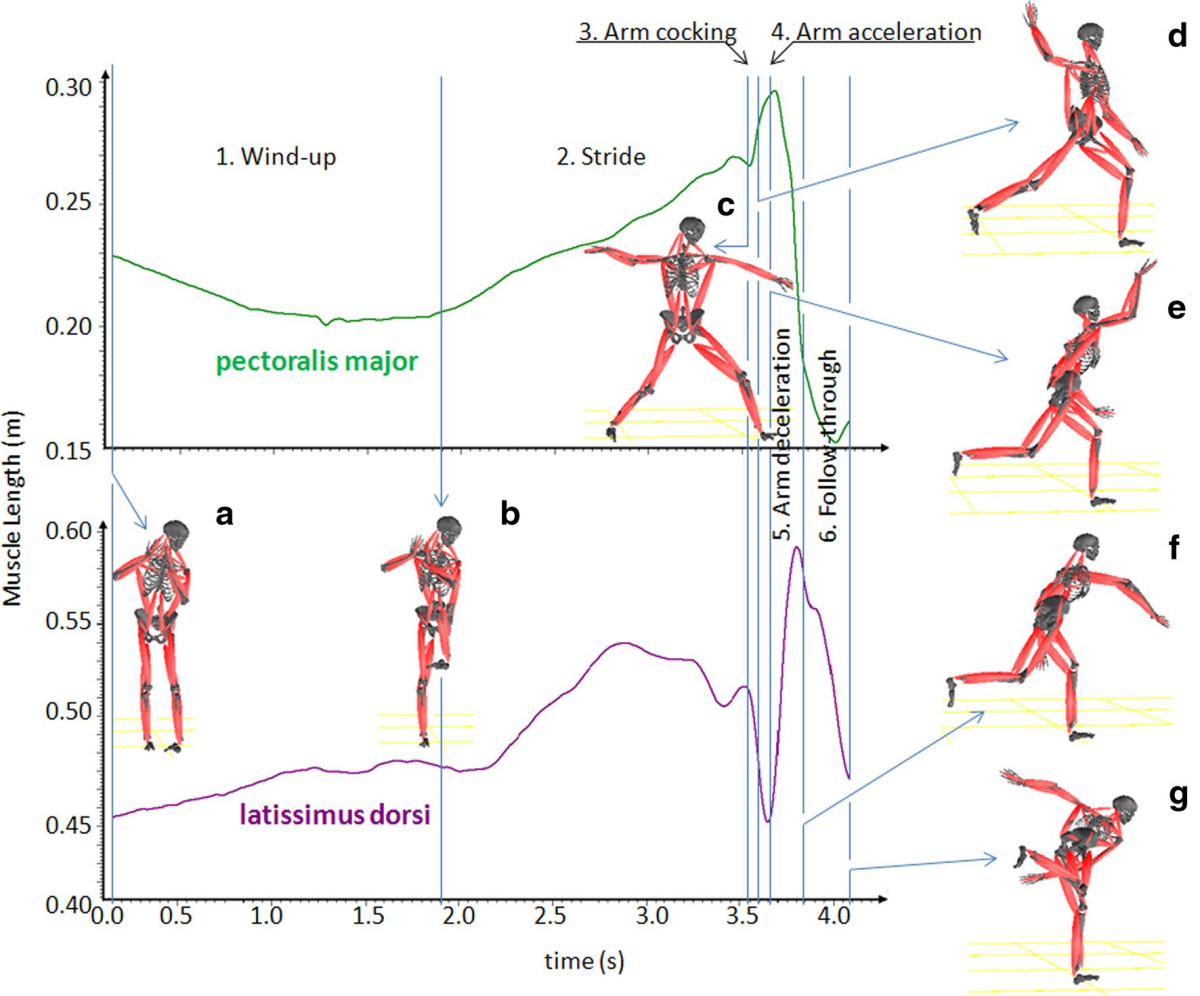
Dynamic lengthening characteristics of pectoralis major and latissimus dorsi during pitching

The current study has successfully unveiled the relationship between the dynamic lengthening characteristics of the two major trunk muscles and the seven events as well as the six phases (Fig. 3). The relationship has supplied direct evidences for studying the injury mechanism of latissimus dorsi.

The results has revealed that, of the six phases, the arm-cocking, arm acceleration, and arm deceleration are the phases when the fastest muscles lengthening, as such high magnitudes of forces and moments are experienced (Fig. 3). The modeling results confirm that an over-lengthening of pectoralis major happens during the arm-cocking, reaching in average 129.3 % of its rest length (129.3 ± 6.2 %). After arm-cocking, the muscle experiences a rapid concentric shortening during the arm-acceleration phase, a perfect example of SSC (stretch–shortening cycle) for increasing muscle power (Komi 2000). These results suggest that the pre-lengthening of pectoralis major would powerfully rotate the pitching arm inwards, horizontally across the body, and outwards in front of the body. Therefore, the energy transfer from the pitcher to the ball relies heavily on the pectoralis major. Even though the maximal lengthening of pectoris major is over the critical value of RSIs (>120 % of its rest length), it is hard to find any injury report in literature. The main reason could be the muscle contraction pattern—concentric—as well as strengthen of the muscle, i.e. one of the strongest human muscles.

Further, the results have showed that, during the three quick-action phases (i.e. arm-cocking, arm acceleration and arm deceleration), pectoralis major and latissimus dorsi coordinate as an agonist and an antagonist (Fig. 3). First, the pre-lengthening of pectoralis major during arm-cocking is supported by the concentric shortening of latissimus dorsi; then, the two muscles exchange their role during the arm-acceleration phase, namely, the explosive, concentric contraction of pectoralis major (<100 ms) is accompanied by a rapid lengthening of latissimus dorsi. Empirical evidence has shown that an imbalance between agonists and antagonists plays an important role in the etiology of muscle injuries (Bejjani 1987). Hence, improving the coordination efficiency between these two major muscles should be emphasized during training. Coaches and trainers should be aware that muscle tightness and soreness would influence muscles coordination, as such, could result in an imbalance between agonists and antagonists (Bejjani 1987; Schickendantz et al. 2009). A study done by Carvalho et al. (2012) shows that, comparing passive and active stretching, 5-min dynamic stretching is the most efficient way to improve performance by enhancing muscle motor units excitability as well as to gain the greatest central activations of motor neuron for improving coordination between agonists and antagonists. Therefore, muscle tightness can be minimized by having dynamic muscle warm-up exercise and individual muscle soreness requires different recovery time, i.e. individual training time.

The purpose of arm deceleration is to slow down the throwing arm comfortably, safely to dissipate the excess kinetic energy that has not been transferred to the ball. As such, the remaining kinetic energy would be a dominant cause for an injury (Pappas et al. 1985; Park et al. 2002). The results of current study have indicated that the quick deceleration (6216°/s ± 218°/s) occurs during an interval of less than 100 ms following the ball release and produces impact-like (speedy increase), eccentric tension on the latisssimus dorsi (Fig. 3). Even at low intensity, the repetitive impact-like loading has been proved to contribute arm-muscle RSIs among musicians (Shan and Visentin 2003); therefore, this type of loading should be dangerous for pitchers, too. Additional to the impact-like, eccentric loading, the muscle also undergoes its over-lengthening in this phase (130.5 ± 7.1 % of its rest length), 10 % over the critical value of RSIs (Faulkner et al. 1993; Fritz and Stauber 1988; McCully and Faulkner 1985; Stauber et al. 1988). All the conditions—impact-like, high-intensity and eccentric over-lengthening—could work together to amplify the injury risk. Such a load is further intensified among professional players as their deceleration approached up to 7000°/s (Braun et al. 2009; Feltner and Dapena 1986; Pappas et al. 1985), comparing to 6216°/s in current study. Collectively, the results suggest that, after ball release, the pectoralis major loses its mechanical advantage; thus, of the two main muscles, the latissimus dorsi is more active than the pectoralis major during the deceleration phase; and the deceleration is mainly achieved by the impact-like, powerful eccentric over-lengthening of the latissimus dorsi, resulting in a highly dangerous loading pattern. In short, the over-lengthened and forceful impact-like eccentric muscle loading should be the cause of latissimus dorsi injury. The injury mechanism would imply that involving stretching/flexibility practice and eccentric loading exercise into regular training programs plays an important role in the prevention of such an injury. Stretching/flexibility practice would increase muscle lengthening ability and eccentric loading exercise could strengthen muscles against a forceful impact-like eccentric loading.

### Fan kick and axe kick

The last aim of this study was to draw possible prevention strategies from the comparison of kinematically similar motor skills with significantly different injury rates. Dancing and martial arts were chosen. Previous studies have indicated that, 64–80 % of injuries among dancers are in the lower extremities (Allen et al. 2012; Arendt and Kerschbaumer 2003; Hincapié et al. 2008; Milan 1994) and muscle strains and tears represent most of the injuries (Allen et al. 2012; Bejjani 1987). One casual factor could be a frequent practice of over-extended dance postures such as fan kick that put repetitively enormous strain on certain muscles. Similar postures, e.g. axe kick, can be found in martial arts. Using 3D motion capture and biomechanical modeling, the current study quantitatively compared the ROM and the muscle dynamic lengthening characteristics of the both kicking skills.

**Fig. 4:**
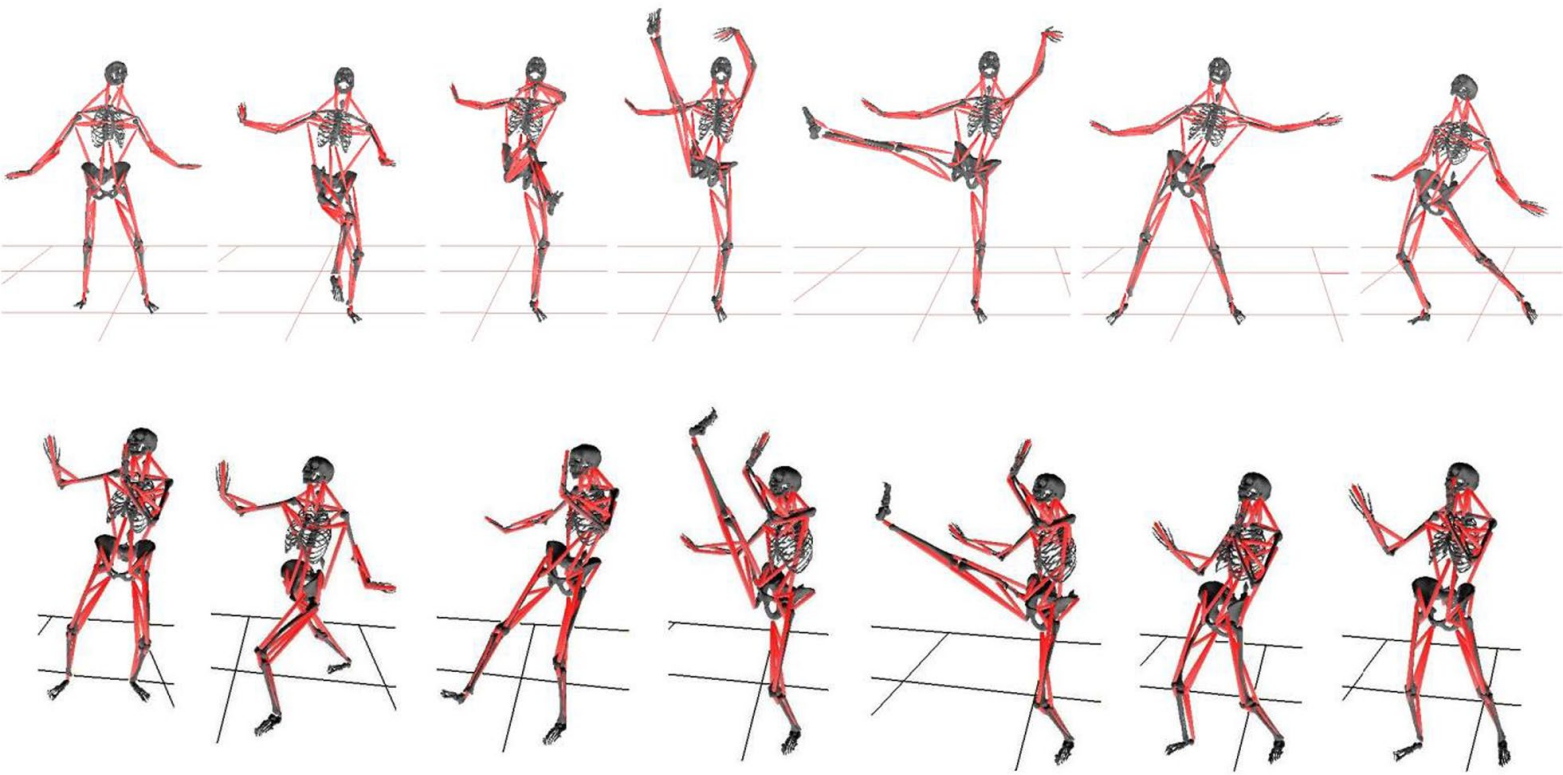
Kinematic similarity between fan kick in dance and axe kick in martial arts

The modeling results (Fig. 4) have showed that the relative leg-positions of the fan kick and the axe kick are quite similar. The former is performed by lifting the working leg across the body to a peak height straight in front of the body and then swinging the extended leg out to the side of the body and downward, while the later skill is conducted by lifting the kick-foot heel with extended leg to its peak height and then bringing the lifted leg straight down with a powerful motion. Since the extremely change in both skills is found in the kick leg, the analyses, comparisons and discussions in this study are purposely selected to focused on the kick-leg hip, knee and major muscles related to the control of these two joints.

The 3D analyses (Table 3) has divulged that the ROM of hip flexion/extension of the kick-leg during an axe kick is significantly larger than that of the fan kick (p < 0.05), reaching an average of 162.2° (162.6° ± 8.4°), whereas the fan kick is 146.3° (146.3° ± 6.6°). However, due to a lateral swing of the kick leg during the fan kick, the ROM of hip abduction/adduction shows a reverse trend, i.e. the value of axe kick (87.8° ± 5.6°) is significantly smaller (p < 0.01) than that of fan kick (51.6° ± 6.1°). There is no difference for hip rotation. Comparison between knees’ ROMs indicates that fan kick experiences significantly larger flexion/extension than that of axe kick for the kick leg (p < 0.01). The values reach 108.7° (108.9° ± 6.3°) for the fan kick, while it is only 65.3° (65.3° ± 4.7°) for the axe kick. There are no significant differences of knee abduction/adduction and rotation between both skills.

**Table 3:** Comparisons of the hip and knee ROMs of the kick leg and the lengthening and lengthening speed of selected kick-leg muscles in both skills (fan kick vs. axe kick)

Joint	ROM (°)		Muscle lengthening				
			Muscle	Max lengthening (% of its rest length)		Lengthening speed (m/s)	
	Fan kick	Axe kick		Fan kick	Axe kick	Fan kick	Axe kick
Hip							
Flex/Ext	146.3 ± 6.6	***162.2*** **±** ***8.4***	Rect Fem	104.2 ± 4.1	105.9 ± 5.0	0.26 ± 0.02	***0.48*** **±** ***0.05***
Abd/Add	***87.8*** **±** ***5.6***	51.6 ± 6.1	Vast Lat	109.5 ± 4.5	103.6 ± 3.9	0.09 ± 0.00	***0.21*** **±** ***0.01***
Rot	54.1 ± 4.1	57.9 ± 5.3	Bi Fem	*127.3* ± *5.2*	*132.0* ± *4.7*	0.44 ± 0.03	***0.69*** **±** ***0.05***
Knee							
Flex/Ext	***108.9*** **±** ***6.3***	65.3 ± 4.7	Add Mag	***197.7*** **±** ***5.3***	*171.3* ± *4.9*	0.42 ± 0.04	***0.61*** **±** ***0.04***
Abd/Add	27.4 ± 4.6	28.8 ± 4.1	Gracilis	*134.7* ± *4.8*	*133.8* ± *3.6*	0.13 ± 0.00	***0.46*** **±** ***0.05***
Rot	28.2 ± 4.1	21.5 ± 4.3	Glut Med	***127.9*** **±** ***4.0***	116.3 ± 3.7	0.27 ± 0.02	0.29 ± 0.02
			Semi	*129.2* ± *4.4*	*133.8* ± *4.3*	0.50 ± 0.03	***0.71*** **±** ***0.06***

Italics: muscles in risk

Bold italics: the value is significantly high (p < 0.05) than that of the counterpart

*Flex/Ext* flexion/extension. *Abd/Add* abduction/adduction, *Rot* rotation

*Rect Fem* rectus femoris, *Vast Lat* vastus lateralis, *Bi Fem* biceps femoris, *Add Mag* adductor magnus, *Glut Med* glutaeus medius, *Semi* semimembranosus

**Fig. 5:**
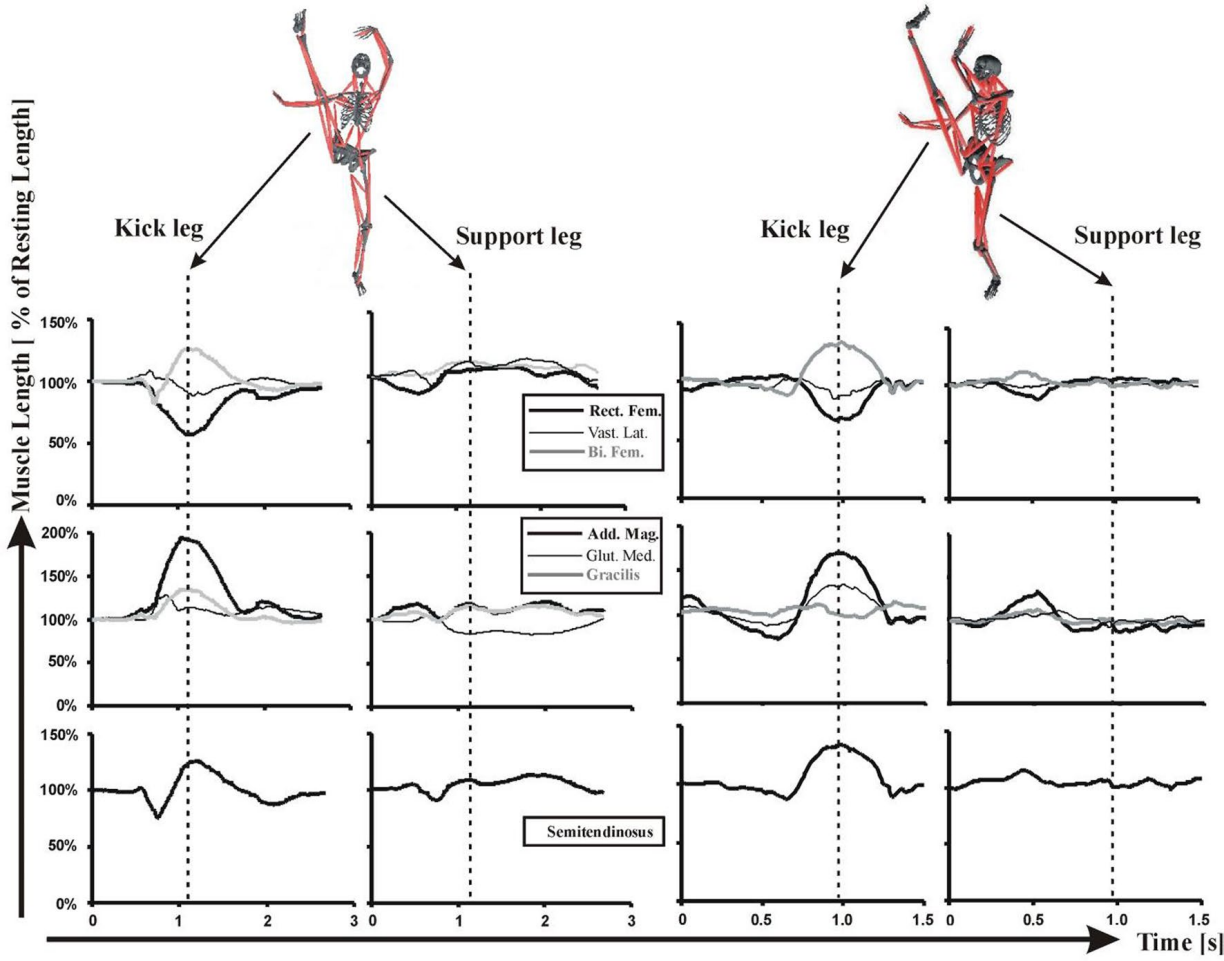
Typical lengthening of selected muscles during fan kick (*left*) and axe kick (*right*)

Looking at muscle lengthening, the modeling results show that these two skills are particularly alike (Fig. 5). The muscle length analysis has confirmed an asymmetric working pattern in which the kick and support leg muscles lengthening undergo, i.e. the kick leg more, the support leg less. As such, the over lengthened muscles are only found on the kick side. For the axe kick, the following four muscles have been proved to be over the critical value of RSIs (>120 % of its rest length): biceps femoris, adductor magnus, gracilis and semimembranosus. The average lengthening for these muscles are: biceps femoris = 132.0 ± 4.7 %, adductor magnus = 171.3 ± 4.9 %, gracilis = 133.8 ± 3.6 %, and semimembranosus = 133.8 ± 4.3 % (Table 3). For the fan kick, in addition to the above mentioned four muscles, the lengthening of right rectus femoris also exceeds the critical value. The over-lengthened muscles during the fan kick have the following values: biceps femoris = 127.3 ± 5.2 %, adductor magnus = 197.7 ± 5.3 %, gracilis = 134.7 ± 4.8 %, rectus femoris = 127.9 ± 4.0 %, and semimembranosus = 129.2 ± 4.4 %. Generally speaking, doing fan kick one experiences more muscle stress than performing axe kick, because two of the over-lengthened muscles (adductor and rectus femoris) during the fan kick are significantly larger than their counterparts during the axe kick (p < 0.00 and p < 0.05 respectively).

A previous study has shown that kick power, as such the loading to muscles, are highly related to muscle lengthening speed and muscle contraction time (Shan et al. 2005). Comparing the fan kick to the axe kick, the latter is significant faster that the former (Table 3; Fig. 5). The muscles’ lengthening speeds of the axe kick are from 50 % to over two times higher than those of the fan kick, while the performance time of the axe kick is about half of that of the fan kick. Collectively, these results suggest that the intensity of muscle loading would be considerably higher during the axe kick than that during the fan kick.

With a larger ROM in hip flexion/extension and a higher muscle loading intensity, the martial arts practitioners would have higher risks of RSIs than dancers; or at least, have an equivalent risk level. However, the reality contradicts the theoretical hypothesis. Why could the higher risk group have a lower injury rate? A close look at the influencing factors as well as learning and training practice of both skills may provide some explanations.

Previous studies (Hincapié et al. 2008; Shan 2005; Twitchett et al. 2009) suggest that the following training-related factors could be related to the RSIs: ROMs of joints, muscle loading pattern (concentric or eccentric), degree of muscle’s lengthening, loading intensity, loading frequency, duration of practice, recovery time, nutrition and loading adaptation through training. Among these factors, frequency, duration, strength (loading intensity) training, recovery time, nutrition and loading adaptation could reverse the injury rate for martial artists. Empirical data show that dancers often repeat more over-lengthening exercise, such as fan kick, than martial artists exercise the axe kick in a training session. In addition, it is also believed that dancers spend more time on skill training, and less time on strength training than martial art practitioners. The ground for generally avoiding fitness training in dance practice could be the tradition and a reluctance to follow principles associated with sport. As a result, dancers often demonstrate low levels of strength even though it is necessary to meet the required workload. Additionally, it is common that dancers perceive strength training leads to aesthetically undesirable hypertrophy. Given that training does not provide the opportunity to expend enough energy to maintain these aesthetic demands, this aesthetic demand may be met by diet restriction, which may lead to subsequent increased injury risk. Because there are no statistical studies available to compare the schedule details of learning and/or training programs between dancing and martial arts, further studies are needed to confirm the conclusion. If this aspect is confirmed by future studies, “rescheduling” current practice plans in the dance learning and/or training will be required to reduce RSIs. The reschedule should include greater recovery time, more strength training, individualized length of practice session and proper diet. Such measures would lead to a “fit for purpose” body (Twitchett et al. 2009), which can help increase the training efficiency, improve performance and reduce RSIs.

### Limitation and future direction

Inevitably, there are limitations in this study. Motor skills in sports vary in a large range. Therefore, the muscles in risk may be different depend on specific sports. Further studies using biomechanical modeling are needed to investigate more motor skills in order to establish a data bank, showing a possible relationship between muscles in risk and sports participated as well as the mechanism of the possible injuries. Such information would help coaches/practitioners improve their training program for minimizing the RSIs in motor skill learning and training.

## Conclusions

The current study explore the potential use of biomechanical modeling in the prevention of muscle injuries related to repetitive movement during complex motor skill learning and training in youth sports. The study aimed to (1) reveal the ROM characteristics and the dynamic muscles lengthening of selected motor skills, (2) identify muscles at risk (lengthening >120 % of its resting length), and (3) compare kinematics of similar skills with different injury rates in order to provide insight into risk assessment and muscle injury prevention. The results of the 4 selected motor-skill studies have shown that the quantification of ROM, muscles lengthening, and the skills comparison help us reveal factors leading to RSIs. Specifically, the results have unveiled that repetitive over-lengthening and impact-like eccentric loading on muscles are the primary casual-factors of RSIs. Muscle stiffness during learning, practice frequency, duration and strength of loading as well as recovery time could jointly contribute to the development of RSIs (i.e. micro-trauma accumulation). Based on the results, measures for reducing the risk of such injuries related to complex skills’ learning and training in youth sport could be: (1) identifying muscles at risk among popular youth physical activities through biomechanical modeling; (2) targeted stretching training of muscles at risk in order to increase lengthening ability; (3) dynamic muscle warming-up before practice for minimizing possible imbalance between agonists and antagonists; (4) limiting practice times of the frequency and duration of intense movements during training with the aim of reducing micro-trauma accumulation; (5) involving more targeted eccentric-loading training on muscles at risk to raise muscles’ capacity against impact-like eccentric loading and (6) allowing enough repair time for recovery from micro-traumas induced by repetitive training (individual training time). Collectively, the results show that biomechanical modeling is a practical tool for predicting injury risk and provides an effective way to establish an optimization strategy to counteract the factors leading to muscle repetitive stress injuries during motor skill learning and training.

## Methods

### Subjects

In order to understand the development of muscle overuse injuries, a quantitative determination of kinematic and muscle lengthening characteristics of standard performances of the selected skills is required. Therefore, well-trained subjects were recruited for unveiling the standard characteristics of the skills in order to assess the muscles at risk during the learning and training of sports skills among youths.

For soccer kicking Investigation, there were 22 advanced players. The subject group consisted of equal number of male and female from college varsity soccer teams, aging from 19 to 26 years with soccer training varying from 11 to 15 years. For baseball pitching exploration, 9 male players of versatile elite-level with 10–16 years of training were recruited. They were between 20 and 25 years old. And for the comparison study, 7 dancers (1 male, 6 females) and 13 Tae Kwon Do athletes (9 males and 4 females) participated in the research project. All the subjects of the comparison study were highly-trained with experience range from 13 to 32 years and age from 23 to 40 years. No subject was experiencing muscle injuries during the time of the study.

The Human Subjects Research Committee of the host university scrutinized and approved the protocols as meeting the criteria of ethical conduct for research involving humans. All subjects were informed of the testing procedures. They signed an approved consent form and voluntarily participated in the data collection.

## 3D motion capture and biomechanical modeling

**Fig. 6:**
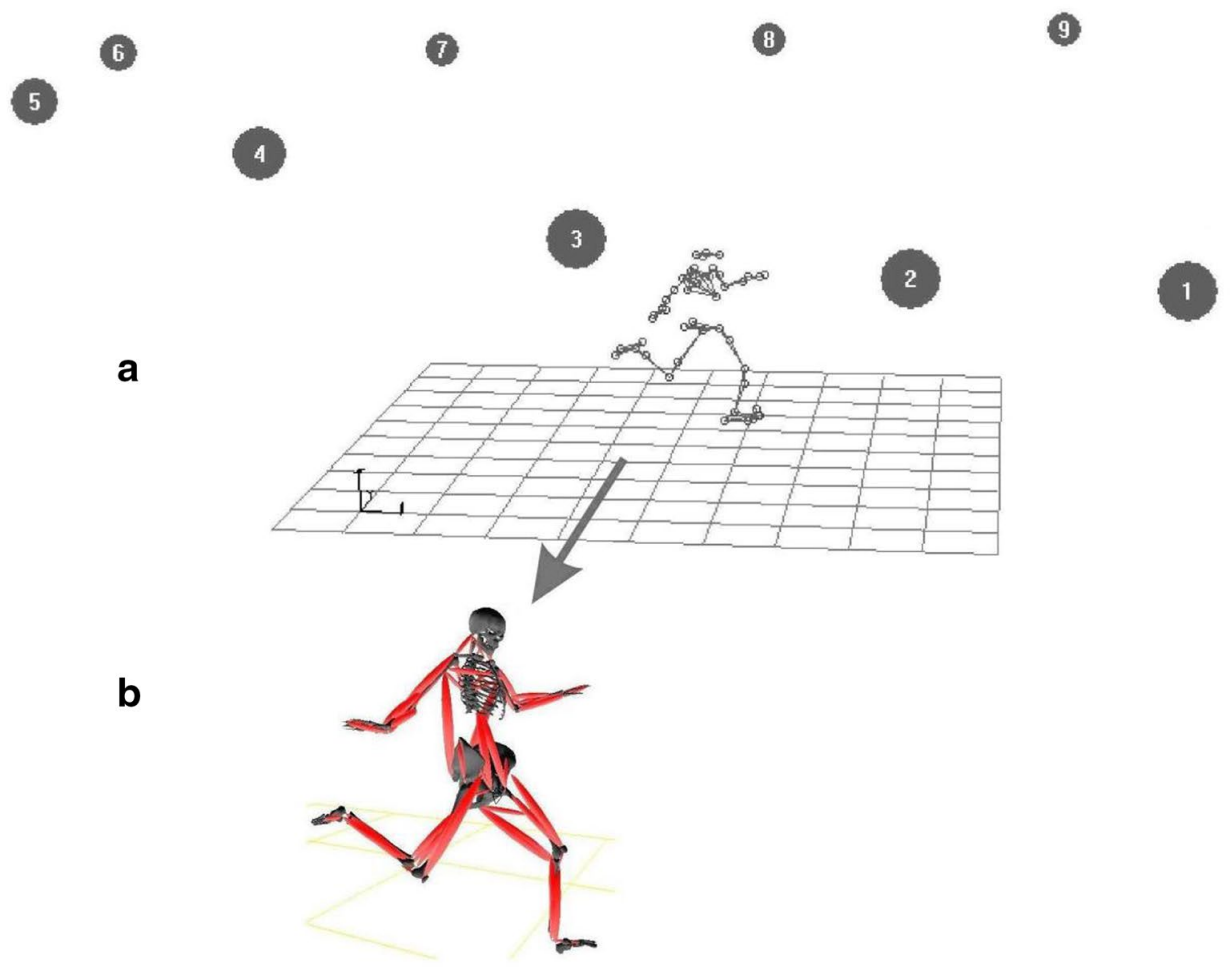
**a** 3D motion capture set-up (nine high-speed cameras), and **b** subject reconstruction (biomechanical model)

A nine-camera, 3D motion-capture system (VICON MX40, Oxford Metrics Ltd., Oxford, England) was used to track 42 reflective markers (9 mm in diameter) on a subject’s body at a rate of 200 frames/s. Markers were placed on subjects as follows: on the head (4), sternal end of the clavicle, xiphoid process of the sternum, C7 and T10 vertebrae, left and right scapula, anterior superior iliac and posterior superior iliac, left and right acromion, lateral side of each upper arm, lateral epicondyles, lateral side of forearms, styloid processes of radii and ulnae, distal ends of 3rd metacarpal bones, left and right lateral sides of thighs and shanks, lateral tibial condyles, lateral malleoli, calcanei, tuberosity of the fifth metatarsal and big toes. Figure 6a shows a 3D computer reconstruction of the capture set-up. The use of nine cameras and small markers permitted considerable freedom of movement for the subjects, ensuring subjects’ movements within the capture volume remained as close to their normal “style” as possible. Calibration residuals were determined in accordance with VICON’s guidelines and yielded positional data accurate within 1 mm. The use of 3D motion capture supplied information, such as distances, velocities, and accelerations of the 42 markers.

From motion capture, one can obtain marker/anatomical positions which allow the modeling of the skeletal structure. From changes in position over time, simple positional data can be translated into skeletal movement using the fundamental precepts of physics. This process is known as biomechanical modeling. A 15-segment, full-body biomechanical model was built by using the 3D capture data. The 15 segments were head, 2 segments trunk, upper arms, lower arms, hands, thighs, shanks and feet. The model allows the calculation of joints’ flexion/extension, abduction/adduction and rotation. The three component can characterize the kinematics of a skill, such as the maximum and minimum of a joint angle and its ROM (i.e. ROM = maximum − minimum). Additionally, muscles can be attached to the skeletal frame using anatomical knowledge regarding attachment points (Fig. 6b). Thus, dynamic muscle lengthening or shortening can be determined in connection with skeletal movement. So do the muscular work patterns (eccentric, concentric, isometric). In such biomechanical modeling, inertial characteristics of the body were estimated using anthropometric “norms” found through statistical studies (Shan and Bohn 2003).

### Data collection and analysis

Subjects were allowed to perform a sufficient number of warm-ups in order to become accustomed to the test environment. The readiness of the warm-up was based on subject’s own feeling. After warm-up, subjects executed three times of his/her selected skill. Each subject decided on his/her own pace between warm up and tests, so that the optimal individual control state would be reached.

Raw 3D data was processed using a five-point (1-3-4-3-1 function) smoothing filter and then, the filtered data was input into the 15-segment model for biomechanical calculation. Descriptive statistics (averages and standard deviation) was performed using Microsoft Excel (2010) to reveal the selected skills’ characteristics; and independent t test was applied to determine possible significant differences (fan kick vs. axe kick) of muscles’ lengthening in order to assessing the injury risk. The level of significance was set at 0.05 for the t tests.
